# Predicting Lymph Node Metastasis in Rectal Cancer: Development and Validation of a Machine Learning Model Using Clinical Data

**DOI:** 10.2196/73765

**Published:** 2025-09-23

**Authors:** Wei Hou, Chuangwei Li, Zhen Wang, Wanqin Wang, Shouhong Wan, Bingbing Zou

**Affiliations:** 1 Department of General Surgery, The First Affiliated Hospital of Anhui Medical University, Hefei, Anhui, 230022, China; 2 Institute of Artificial Intelligence, Hefei Comprehensive National Science Center, Hefei, China; 3 Anhui Medical University, Hefei, China; 4 Department of Radiology, The First Affiliated Hospital of Anhui Medical University, Hefei, China; 5 School of Computer Science and Technology, University of Science and Technology of China, Hefei, China

**Keywords:** rectal cancer, lymph node metastasis, machine learning model, prediction models, clinical data, prognosis, XGBoost

## Abstract

**Background:**

Rectal cancer (RC) is a common malignant tumor, with lymph node metastasis (LNM) being a critical determinant of patient prognosis. Traditional diagnostic methods have limitations, necessitating the development of predictive models using clinical data.

**Objective:**

This study aimed to construct and validate machine learning (ML) models to predict LNM risk in patients with RC based on clinical data.

**Methods:**

Retrospective data from 2454 patients with RC (SEER [Surveillance, Epidemiology, and End Results] database) were split into training (n=1954) and internal validation (n=500) sets. An external cohort (n=500) was obtained from the First Affiliated Hospital of Anhui Medical University. Lymph node features identified via computed tomographic scans were integrated with clinicopathological data. Variables were selected using LASSO (Least Absolute Shrinkage and Selection Operator), followed by univariate and multivariate logistic regression. Eleven ML models (Logistic Regression, K-Nearest Neighbors, Extremely Randomized Trees, Naive Bayes, XGBoost [XBG], Light Gradient Boosting Machine, Multilayer Perceptron, Gradient Boosting, Support Vector Machine, Random Forest, and Ada-Boost) were evaluated via area under the receiver operating characteristic curve (AUC), calibration curves, and decision curve analysis.

**Results:**

LNM prevalence was 26.9% (training), 27% (internal validation), and 81% (external validation). Independent LNM predictors included tumor grade, clinical T stage, N stage, tumor length, neural invasion, and total lymph nodes. Internal validation AUC ranged from 0.859 to 0.964; external validation AUC was 0.735‐0.838. In the internal validation set, Random Forest and Extremely Randomized Trees achieved the highest AUC (0.964, 95% CI 0.950‐0.978), while XGB demonstrated superior cross-cohort stability (AUC 0.942, 95% CI 0.925‐0.959). For external validation, Gradient Boosting had the highest AUC (0.838, 95% CI 0.801‐0.875), followed by XGB (0.832, 95%CI 0.794‐0.869). XGB showed minimal calibration error with curves closest to the ideal diagonal and yielded the highest net benefit in decision curve analysis across critical thresholds.

**Conclusions:**

This study successfully developed and validated 11 ML models to predict LNM risk in RC. The XGB model was optimal, achieving an AUC >0.9 in 10 internal models and an AUC >0.8 in 7 external models. The identified predictors of LNM can facilitate early diagnosis and personalized treatment, highlighting the potential of integrating computed tomographic scan data with clinicopathological findings to build effective predictive models.

## Introduction

Rectal cancer (RC) is among the most prevalent malignant tumors globally and is currently the second leading cause of cancer-related deaths worldwide [1,2]. A recent study [3] projects that by 2040, the incidence of RC will rise to 3.2 million new cases annually, with 1.6 million deaths worldwide. Lymph node metastasis (LNM) is a critical determinant of poor prognosis in RC, and numerous studies [4-7] have shown that accurate prediction of LNM is crucial for treatment selection in patients and prognostic assessment. Initially, while current imaging technologies can assess the risk of LNM to some degree [8-10], they largely depend on radiologists’ anatomical expertise for evaluation [11]. However, these methods still face limitations in terms of accuracy and efficiency [12]. Specifically, conventional computed tomography (CT)–based N staging struggles to detect small metastatic lymph nodes (<5 mm) and subtle morphological changes (eg, irregular borders, heterogeneous texture), which are critical for early metastasis diagnosis but require expert manual annotation [13]. Second, pathologists determine the presence of LNM in patients with RC based on clinical pathology reports. This approach is inefficient and subject to the constraints of the individual pathologist’s expertise.

In recent years, the rapid advancement of machine learning (ML) technology has led to its growing application in the medical field, particularly in disease prediction, diagnosis, and treatment decision-making, demonstrating significant potential [14,15]. ML algorithms can process and analyze large volumes of clinical data, identifying disease-related patterns and features, which enhances the accuracy of predictive models [16]. However, most existing ML models rely on automated radiomic features or clinical data alone, neglecting the value of radiologist-annotated CT morphological features [17]. In predicting LNM in RC, previous studies have utilized ML models, including Support Vector Machines, neural networks, and decision trees, achieving notable results [18-21]. Both clinical data and CT results are not entirely accurate in predicting pathological LNM. In the study by Li et al [22], the overall accuracy of N stage based on CT images ranged from 59% to 68%. Meanwhile, up to 70% of metastatic lymph nodes in colorectal cancer have a diameter of less than 5 mm. This indicates that both clinicopathological features and preoperative CT have certain limitations in predicting the malignant lymph node status of patients with T1 colorectal cancer. Therefore, there is an urgent need to develop novel predictive tools that integrate ML with radiological parameters and clinical data, thereby enhancing the accuracy and efficiency of diagnostic processes. Recent evidence suggests that systematic manual annotation by trained radiologists can improve the sensitivity of detecting sub-centimeter metastatic lymph nodes compared with routine clinical reports. This highlights the potential of this method in bridging current diagnostic gaps [23].

This study aimed to develop and validate ML models using clinical data to predict LNM risk in RC. We successfully constructed and validated 11 predictive models integrating radiologist-annotated CT lymph node features with clinicopathological data. These models show significant potential to assist clinicians in early diagnosis and personalized treatment planning. We anticipate that the findings will provide new insights for RC management and serve as a valuable reference for future research and practice.

## Methods

### Data Collection and Inclusion Criteria

This study encompassed clinical data from 2454 patients with RC in the SEER database, wherein 1954 cases were randomly selected to form the training cohort, and the remaining 500 formed the internal validation cohort. Additionally, it included data from 500 patients with RC treated at the author’s hospital between January 1, 2017, and December 31, 2023, which served as the external validation cohort. The inclusion criteria were (1) patients with RC staged as I-IV according to the American Joint Committee on Cancer (AJCC) staging system; (2) those who underwent curative surgery; and (3) those with complete clinical and pathological information; and (4) radical surgery in stage IV patients. In this study, some patients with stage IV RC with oligometastasis (limited resectable metastases) or locally advanced tumors with resectable metastatic lesions underwent radical surgery after evaluation by a multidisciplinary team. The surgical decision was based on the multidisciplinary team’s comprehensive assessment of tumor burden, physical condition, expected survival, and surgical risks. The goal was to achieve R0 resection and combine it with postoperative adjuvant therapy to reduce recurrence risk. Data for these patients were complete and underwent strict screening to ensure they met the research criteria. The exclusion criteria included (1) a history of other malignant tumors, (2) inability to assess lymph node status, (3) incomplete clinical information, and (4) administration of neoadjuvant therapy. The LNM status for 2454 patients with RC in the SEER database was ascertained from pathological assessments of surgical specimens. For the 500 cases at the author’s hospital, LNM status, clinical T and N staging, and tumor length were determined by precisely annotating and measuring contrast-enhanced CT images with the radiological software ITK-SNAP software (version: 4.0.1; Professor Paul Yushkevich's team, University of Pennsylvania, USA), corroborated by pathological assessments from surgical specimens. For example, the definition of tumor length in the SEER database (training/internal validation cohorts) was measured from pathological specimens. In the external validation cohort, tumor length was annotated and measured on contrast-enhanced CT scans using ITK-SNAP software by radiologists, with cross-validation against postoperative pathology (mean error <0.3 cm).

### Clinical Pathological Features

**Figure 1.**
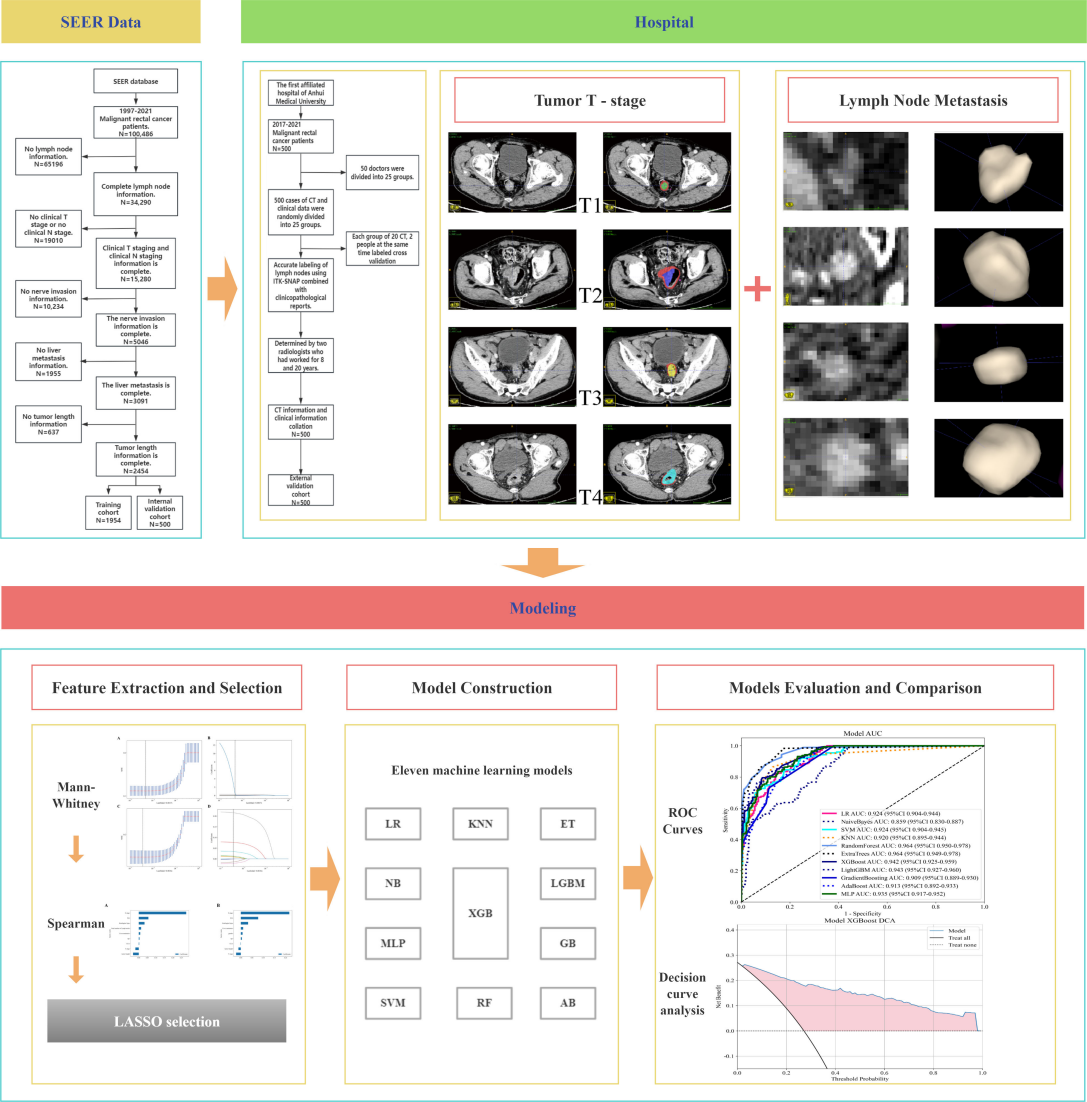
Workflow of this study. AB: Ada-Boost; CT: computed tomography; ET: Extremely Randomized Trees; GB: Gradient Boosting; KNN: K-Nearest Neighbors; LGBM: Light Gradient Boosting Machine; LR: Logistic Regression; MLP: Multilayer Perceptron; NB: Naive Bayes; RF: Random Forest; ROC: receiver operating characteristic; SEER: Surveillance Epidemiology and End Results; SVM: Support Vector Machine; XGB: XGBoost**.**

The study collected the following clinical and pathological characteristics of the patients: sex, age, perineural invasion, carcinoembryonic antigen (CEA), tumor length, clinical T stage, N stage, tumor differentiation, chemotherapy and radiotherapy administration, liver metastasis status, and tumor histology. For the training and internal validation cohorts, data extraction was conducted using the SEER*STAT software, version 8.4.3 (Surveillance, Epidemiology, and End Results Program, National Cancer Institute, USA). In the external validation cohort, clinical data were independently collected and processed by 2 researchers, while CT images were annotated and measured with precision by 50 physicians, evenly distributed into 25 groups, each consisting of 2 physicians for cross-validation purposes. The inclusion criteria for metastatic lymph nodes were fulfilled if any of the following five conditions was met: (1) the short axis/long axis ratio was ≥0.8; (2) the short axis was ≥5 mm; (3) there was aggregation of three or more lymph nodes; (4) the lymph node had an irregular shape with a rough margin; (5) the lymph node signal was inhomogeneous, with high signal areas in the CT imaging for lymph nodes (including mesenteric and presacral lymph nodes). Figure 1 illustrates the acquisition process.

### Data Processing and Analysis

All collected data will be subjected to rigorous preprocessing in Python, encompassing data cleaning, outlier detection, and missing value imputation. Subsequent statistical analysis will utilize suitable methods including *χ*^2^ tests, ANOVA, and both univariate and multivariate regression analyses to evaluate the correlations between clinical pathological features and LNM.

### Feature Engineering and Selection

In this study, we standardized numerical features, including tumor length, to mitigate the effects of varying scales and enhance their compatibility with the input requirements of ML models. For categorical variables like clinical T staging, we applied label encoding to transform them into numerical data. All features were first normalized to eliminate scale effects (Multimedia Appendix 1). We then performed univariate feature screening via independent *t* tests or Mann-Whitney *U* tests (*P*≤.05), retaining features. Next, Pearson correlation analysis reduced redundancy by removing features with mean absolute correlation >0.9, yielding decorrelated features. Finally, features with nonzero coefficients were selected through LASSO regression (10-fold cross-validated) with regularization parameter α. Notably, this LASSO-based selection served as initial screening; tree-based models (Random Forest [RF]/XGBoost [XGB]/Light Gradient Boosting Machine [LGBM]) subsequently performed endogenous feature reweighting to capture nonlinear interactions during model training. This method incorporates an L1 regularization term that aids in pinpointing the most influential features while promoting model sparsity. Furthermore, we performed a correlation analysis among the features to verify the independence of the selected variables. Utilizing the principle of Permutation Importance, we evaluated the importance of each feature to ascertain its contribution to the model’s predictive performance.

### Predictive Model Construction and Validation

This study developed 11 distinct ML models: Logistic Regression (LR), Naive Bayes (NB), Support Vector Machine, K-Nearest Neighbors (KNN), RF, Extremely Randomized Trees (ET), XGB, LGBM, Gradient Boosting (GB), Ada-Boost (AB), and Multilayer Perceptron (MLP), for predicting the risk of LNM in patients with RC (Multimedia Appendix 2). To counteract the imbalance in data distribution, we used random oversampling techniques and applied a penalty parameter λ to drive the coefficient estimates of nonsignificant features toward zero. The models’ performance was evaluated through 10-fold cross-validation. The performance of each ML classifier was assessed using the receiver operating characteristic curve, where a higher area under the receiver operating characteristic curve (AUC) indicates greater predictive accuracy. We evaluated the variable weights and permutation importance and used heatmaps to visualize the significance and correlations among the variables. To select the optimal model, we evaluated each model’s performance across multiple metrics: AUC (with 95% CI), accuracy, sensitivity, specificity, and F_1_ score. Calibration curves and decision curve analysis (DCA) were further employed to assess calibration accuracy and clinical utility, particularly focusing on net benefits at critical thresholds.

### Ethical Considerations

This study was approved by the Ethics Committee of the First Affiliated Hospital of Anhui Medical University (Approval No.: Quick-PJ 2023-13-34). As a retrospective analysis using anonymized data, the requirement for informed consent was waived. Primary data collection in the SEER database obtained patient informed consent, and its Institutional Review Board explicitly authorized secondary analysis without additional approval. All direct identifiers (including names, ID numbers, medical record numbers) were removed from our hospital’s dataset, with only aggregated data utilized in the analyses to ensure privacy protection. No participant compensation was involved, and no identifiable images appear in the manuscript or supplementary materials.

## Results

### Demographic Characteristics and Parameter Selection

In our study, the training cohort included 1954 patients with RC, the internal validation cohort included 500 patients with RC, and the external validation cohort included 500 patients with RC. Across the training cohort, internal validation cohort, and external validation cohort, stratification by LNM status (positive vs negative) revealed statistically significant differences in age, gender, total lymph node count, tumor length, neural invasion, clinical T stage, N stage, liver metastasis status, tumor histology, and tumor differentiation (all *P*<.001; see Table 1). For CEA, the *P* value was .001, indicating a statistically significant difference (Table 1). We used the LASSO regression method to identify a significant set of risk factors for predicting the risk of LNM in patients with RC.

**Table 1.** The clinicopathological features of the training, internal validation, and external validation cohorts.

Characteristics	Training cohort (N=1954)		Internal validation cohort (N=500)		External validation cohort (N=500)		*P* value
	Negative (n=1421)	Positive (n=533)	Negative (n=365)	Positive (n=135)	Negative (n=195)	Positive (n=305)	
Sex, n (%)							
Female	543 (38.2)	197 (37.0)	193 (5.0)	44 (33.0)	75 (38.0)	101 (33.0)	<.001
Male	878 (62.0)	336 (63.0)	172 (47.0)	91 (67.0)	120 (62.0)	204 (67.0)	
Age, n (%)							
20≤X≤24	2 (0.1)	2 (0.4)	1 (0.3)	2 (1.5)	0 (0)	0 (0)	<.001
25≤X≤29	8 (0.6)	4 (0.8)	6 (1.6)	2 (1.5)	0 (0)	1 (0.3)	
30≤X≤34	20 (1.4)	10 (1.9)	11 (3.0)	3 (2.2)	1 (0.5)	3 (1.0)	
35≤X≤39	36 (2.5)	14 (2.6)	9 (2.5)	6 (4.4)	2 (1.0)	3 (1.0)	
40≤X≤44	63 (4.4)	27 (5.1)	25 (6.9)	18 (13.3)	5 (2.6)	6 (2.0)	
45≤X≤49	97 (6.8)	59 (11.1)	57 (15.6)	16 (11.9)	14 (7.2)	28 (9.2)	
50≤X≤54	181 (13.0)	76 (14.3)	54 (14.8)	19 (14.1)	21 (10.8)	39 (12.8)	
55≤X≤59	211 (15.0)	69 (13.0)	46 (12.6)	17 (12.6)	35 (18.0)	40 (13.1)	
60≤X≤64	216 (15.0)	75 (14.1)	57 (15.6)	12 (8.9)	28 (14.4)	28 (9.2)	
65≤X≤69	217 (15.0)	63 (11.8)	42 (11.5)	15 (11.1)	25 (12.8)	64 (21.0)	
70≤X≤74	164 (12.0)	52 (9.8)	29 (8.0)	10 (7.4)	27 (13.9)	48 (15.7)	
75≤X≤79	107 (8.0)	33 (6.2)	17 (4.7)	10 (7.4)	24 (12.3)	29 (9.5)	
80≤X≤84	63 (4.4)	26 (4.9)	11 (3.0)	5 (3.7)	10 (5.1)	14 (4.6)	
85≤X	36 (2.5)	23 (4.3)	1 (0.3)	2 (1.5)	3 (1.5)	2 (0.7)	
Total number of lymph nodes, n (%)							
0<X≤5	89 (6.3)	17 (3.2)	20 (5.5)	7 (5.2)	3 (1.5)	4 (1.3)	<.001
5<X≤10	130 (9.0)	40 (7.5)	24 (6.6)	10 (7.4)	36 (18.5)	30 (9.8)	
10<X≤15	455 (32.0)	148 (28.0)	113 (31.0)	40 (29.6)	130 (67.0)	211 (69.0)	
15<X≤20	319 (22.0)	131 (25.0)	87 (23.9)	36 (26.7)	23 (11.8)	53 (17.4)	
20<X≤25	186 (13.0)	77 (14.5)	62 (17.0)	15 (11.1)	3 (1.5)	6 (20.0)	
25<X≤30	106 (7.0)	50 (9.4)	28 (7.7)	12 (8.9)	0 (0)	1 (0.3)	
30<X	134 (9.0)	70 (13.1)	30 (8.2)	15 (11.1)	0 (0)	0 (0)	
Tumor length, n (%)							
0<X≤5	883 (62.0)	331 (62.0)	219 (60.0)	81 (60.0)	73 (37.4)	123 (40.0)	<.001
5<X≤10	504 (35.0)	191 (36.0)	138 (38.0)	54 (40.0)	118 (61.0)	168 (55.0)	
10<X	34 (2.4)	11 (2.1)	8 (2.2)	0 (0)	4 (2.1)	14 (4.6)	
PNI^a^, n (%)
Negative	1320 (93.0)	398 (75.0)	339 (93.0)	102 (76.0)	125 (64.0)	176 (58.0)	<.001
Positive	101 (7.0)	135 (25.0)	26 (7.1)	33 (24.4)	70 (35.9)	129 (42.3)	
CEA^b^, n (%)
Negative	933 (66.0)	305 (57.0)	236 (65.0)	77 (57.0)	145 (74.4)	208 (68.0)	<.001
Positive	488 (34.0)	228 (43.0)	129 (35.0)	58 (43.0)	50 (25.6)	97 (31.8)	
Clinical T stage, n (%)							
1	220 (15.0)	37 (6.9)	41 (11.2)	8 (5.9)	22 (11.3)	26 (8.5)	<.001
2	243 (17.1)	70 (13.1)	80 (21.9)	14 (10.4)	62 (31.8)	66 (21.6)	
3	778 (55.0)	339 (64.0)	192 (53.0)	89 (65.9)	92 (47.2)	166 (54)	
4	179 (13.0)	87 (16.3)	52 (14.3)	24 (17.8)	19 (9.7)	47 (15.4)	
N stage, n (%)							
0	879 (62.0)	1 (0.2)	227 (62.0)	0 (0)	116 (59.0)	141 (46.0)	<.001
1	542 (38.0)	394 (74.0)	138 (38.0)	103 (76.0)	50 (25.6)	100 (33.0)	
2	0 (0)	138 (26.0)	0 (0)	32 (23.7)	29 (14.9)	64 (21.0)	
Liver metastasis, n (%)							
Negative	1393 (98.0)	491 (92.0)	361 (99.0)	124 (92.0)	191 (98.0)	301 (99.0)	<.001
Positive	28 (2.0)	42 (7.9)	4 (1.1)	11 (8.2)	4 (2.1)	4 (1.3)	
Histological type, n (%)							
Adenocarcinoma	1378 (97.0)	489 (92.0)	359 (72.0)	126 (25.0)	193 (39.0)	301 (60.0)	<.001
Mucinous /signet ring cell	29 (2.0)	35 (6.6)	5 (1.0)	5 (1.0)	2 (0.4)	4 (0.8)	
Others	4 (0.3)	9 (1.7)	1 (0.2)	4 (0.8)	0 (0)	0 (0)	
Differentiation extent, n (%)							
Poorly differentiated/ undifferentiated	1 (0.1)	0 (0)	0 (0)	0 (0)	10 (5.1)	23 (7.5)	<.001
Moderately differentiated	1421 (100.0)	532 (99.81)	365 (100.0)	135 (100.0)	152 (78.0)	236 (77.0)	
Well-differentiated	0 (0)	0 (0)	0 (0)	0 (0)	33 (16.9)	46 (15.1)	

a PNI: perineural invasion.

b CEA: carcinoembryonic antigen.

### Risk Factors for Lymph Node Metastasis

**Figure 2.**
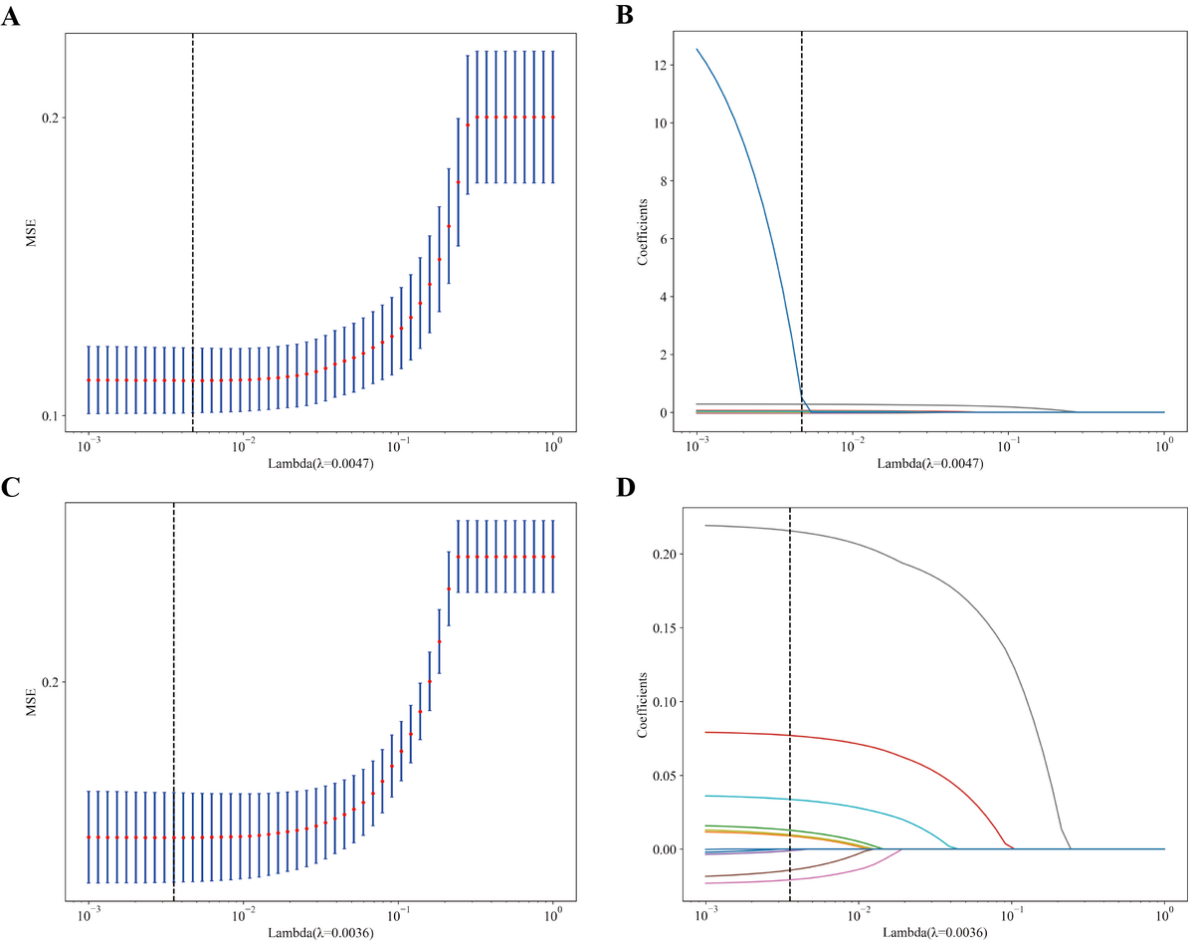
Feature selection using LASSO logistic regression. (A) Adjustment parameters in the LASSO logistic regression for both the training and internal validation sets (C) are selected using 10-fold cross-validation with minimum criteria. The relationship between binomial deviation and the logarithm of the penalty parameter (λ) is depicted. The optimal λ is indicated by a black vertical line, determined by the minimum criterion and the minimum standard error of λ. (B) LASSO coefficients for 12 clinical factors in the training set and internal validation set (D) are presented, illustrating the coefficient profiles against the logarithm of λ. LASSO: Least Absolute Shrinkage and Selection Operator; MSE: mean squared error.

**Figure 3.**
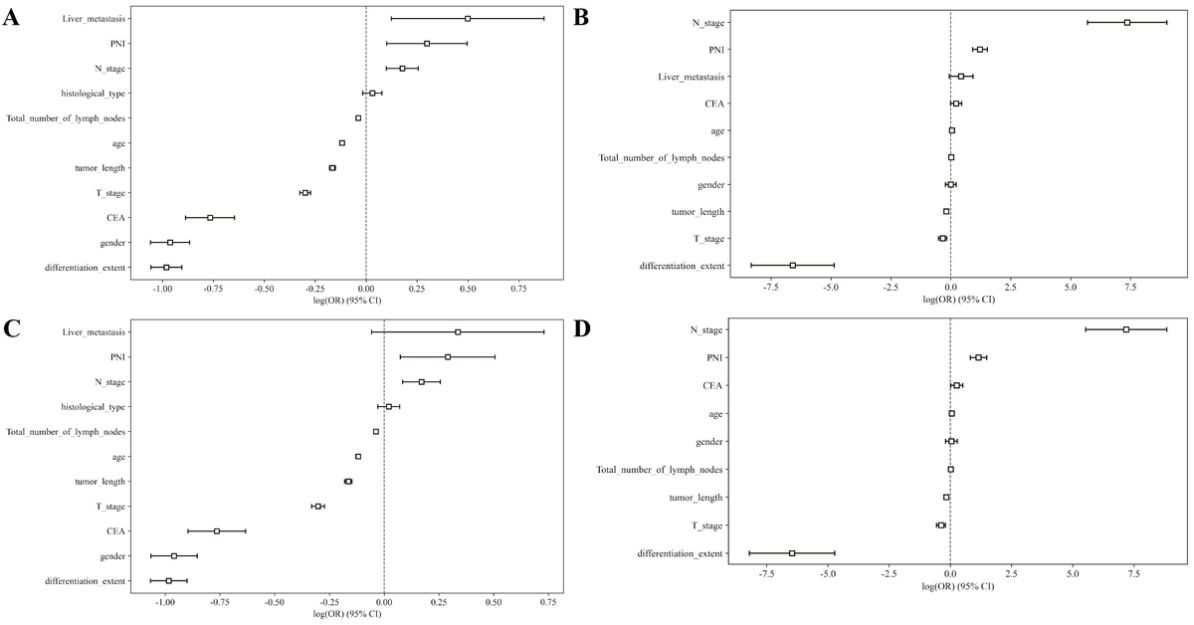
Univariate and multivariate logistic regression analyses yielded a forest plot of odds ratios. (A) In the external validation cohort, single-factor analysis yielded a forest plot of odds ratio. (B) In the external validation cohort, multiple factor analysis yielded a forest plot of odds ratios. (C) In the internal validation cohort, single-factor analysis yielded a forest plot of odds ratios. (D) In the internal validation cohort, multiple factor analysis yielded a forest plot of odds ratios. CEA: carcinoembryonic antigen; PNI: perineural invasion**.**

Using the nonzero coefficients from the LASSO logistic regression model as a guide, we applied both the LASSO method and the multivariate logistic regression model to identify the risk factors associated with LNM in patients with RC, as depicted in Figure 2. The penalty parameters λ for the internal and external validation sets were found to be 0.0047 and 0.0036, respectively. Univariate and multivariate analyses yielded the forest plots for both the internal and external validation sets, as shown in Figure 3. Key predictors from univariate and multivariate analyses are detailed in Table 2. This analysis assists in pinpointing factors that potentially contribute to LNM, marking a pivotal step in comprehending disease progression and in formulating evidence-based treatment strategies. The multivariate logistic regression analysis of the external validation set identified number of peritumoral and total lymph nodes examined (*P*=.022), tumor length (*P*<.001), neural invasion (*P*<.001), clinical T stage (*P*<.001), N stage (*P*<.01), and tumor differentiation grade (*P*<.01) as independent risk factors for LNM.

**Table 2.** Univariate and multivariate logistic regression analysis.

Characteristics	Univariate logistic			Multivariable logistics		
	OR^a^ (CI)		*P* value	OR (CI)		*P* value
Internal validation cohort						
Sex	0.382 (0.347‐0.420)		<.001	0.999 (0.803‐1.244)		.99
Age	0.888 (0.879‐0.896)		<.001	1.046 (1.002‐1.092)		.09
Total number of lymph nodes	0.962 (0.958‐0.966)		<.001	1.017 (1.006‐1.028)		.01
Tumor length	0.847 (0.834‐0.860)		<.001	0.828 (0.786‐0.873)		<.001
PNI^b^	1.347 (1.106‐1.640)		.01	3.372 (2.472‐4.600)		<.001
CEA^c^	0.465 (0.412‐0.524)		<.001	1.245 (0.992‐1.564)		.11
Clinical T stage	0.741 (0.722‐0.761)		<.001	0.711 (0.602‐0.839)		<.001
N stage	1.193 (1.103‐1.290)		<.001	1569.010 (300.065‐8209.112)		<.001
Liver metastasis	1.645 (1.131‐2.392)		.03	1.533 (0.931‐2.522)		.16
Differentiation extent	0.375 (0.347‐0.405)		<.001	0.001 (0.000‐0.008)		<.001
External validation cohort						
Sex	0.383 (0.344‐0.425)		<.001	1.042 (0.821‐1.323)		.78
Age	0.887 (0.878‐0.897)		<.001	1.055 (1.007‐1.105)		.06
Total number of lymph nodes	0.962 (0958‐0.967)		<.001	1.016 (1.005‐1.028)		.02
Tumor length	0.848 (0.834‐0.862)		<.001	0.843 (0.797‐0.891)		<.001
PNI	1.337 (1.077‐1.660)		.03	3.142 (2.246‐4.397)		<.001
CEA	0.465 (0.408‐0.531)		<.001	1.295 (1.012‐1.655)		.09
Clinical T stage	0.740 (0.718‐0.762)		<.001	0.681 (0.568‐0.815)		<.001
N stage	1.185 (1.088‐1.292)		<.001	1307.798 (249.635‐6849.973)		<.001
Liver metastasis	1.400 (0.945‐2.075)		.16	Ref (Ref)		Ref
Differentiation extent	0.374 (0.344‐0.407)		<.001	0.002 (0.000‐0.009)		<.001

a OR: odds ratio.

b PNI: perineural invasion.

c CEA: carcinoembryonic antigen.

### Optimal Predictive Model Selection

#### Feature Weight Ranking

**Figure 4.**
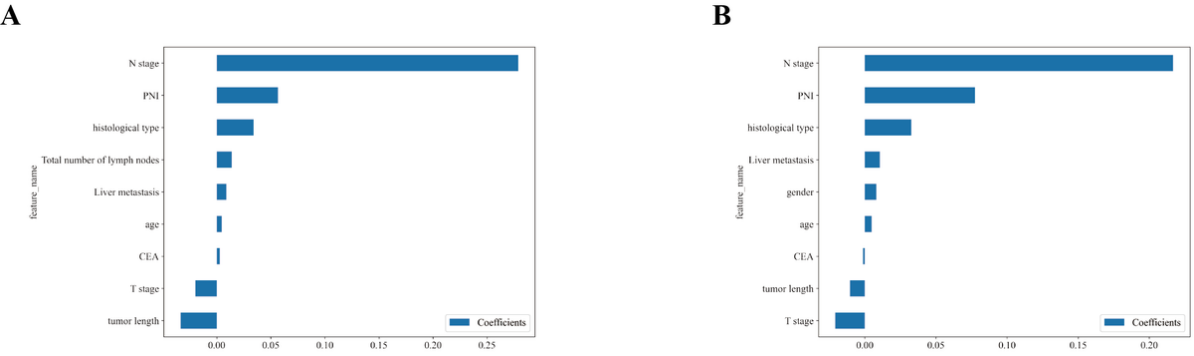
The weight of clinical features in the lymph node prediction model of rectal cancer. (A) Internal validation cohort. (B) External validation cohort. CEA: carcinoembryonic antigen; PNI: perineural invasion.

Based on the penalty coefficient obtained from cross-validation, we selected features with coefficients greater than 0 and represented them using feature weights. The features in the training set and internal validation set are ranked as follows: Clinical N staging, receipt of chemotherapy or radiotherapy, neural invasion, tumor histology, tumor length, number of peritumoral and total lymph nodes examined, liver metastasis, age, CEA, clinical T stage, and tumor length (Figure 4A). In contrast, the features in the training set and external validation set are ranked as follows: Clinical N staging, receipt of chemotherapy or radiotherapy, neural invasion, tumor histology, tumor length, liver metastasis, gender, differentiation degree, age, CEA, tumor length, and clinical T stage (Figure 4B).

#### Model Performance Evaluation

In the internal validation cohort (n=500), the 11 ML models demonstrated a wide range of discriminative performance (AUC range 0.859‐0.964). RF (AUC 0.964, 95% CI 0.950‐0.978) and ET (AUC 0.964, 95% CI 0.949‐0.978) achieved the highest AUC values, with sensitivities of 0.881 and 0.983 and specificities of 0.900 and 0.828, respectively (Multimedia Appendix 3). NB showed the lowest AUC (0.859, 95% CI 0.830‐0.887) despite perfect sensitivity (1.000) but with specificity limited to 0.566. The XGB model yielded an AUC of 0.942 (95% CI 0.925‐0.959), sensitivity of 0.795, and specificity of 0.913. In the external cohort (n=500), Gradient Boosting exhibited the highest AUC (0.838, 95% CI 0.801‐0.875) but the lowest sensitivity (0.685); XGB (AUC 0.832, 95% CI 0.794‐0.869) and LGBM (AUC 0.831, 95% CI 0.793‐0.869) ranked second and third, with XGB demonstrating the smallest sensitivity-specificity gap (*Δ*=0.113) and an *F*_1_-score of 0.690 (Table 3).

**Table 3.** Comprehensive performance metrics of machine learning models for lymph node metastasis prediction in patients with rectal cancer across validation cohorts.

Model name	AUC^a^ (95% CI)		Accuracy	Sensitivity	Specificity	*F*_1_ ^b^
Internal validation cohort						
LR^c^	0.924 (0.9043‐0.9435)		0.849	0.824	0.858	0.747
Naive Bayes	0.859 (0.8300‐0.8873)		0.684	1.000	0.566	0.632
SVM^d^	0.924 (0.9038‐0.9446)		0.801	0.920	0.756	0.715
KNN^e^	0.920 (0.8949‐0.9442)		0.869	0.864	0.871	0.781
Random Forest	0.964 (0.9502‐0.9784)		0.895	0.881	0.900	0.820
Extremely Randomized Trees	0.964 (0.9491‐0.97820)		0.870	0.983	0.828	0.805
XGBoost	0.942 (0.9251‐0.9585)		0.881	0.795	0.913	0.784
LGBM^f^	0.943 (0.9266‐0.9602)		0.872	0.835	0.886	0.780
Gradient Boosting	0.909 (0.8889‐0.9298)		0.722	1.000	0.619	0.662
AdaBoost	0.913 (0.8924‐0.9330)		0.756	0.989	0.669	0.688
MLP^g^	0.935 (0.9168‐0.9525)		0.813	0.932	0.769	0.731
External validation cohort						
LR^c^	0.821 (0.7817‐0.8611)		0.760	0.702	0.789	0.667
Naive Bayes	0.749 (0.7027‐0.7949)		0.662	0.845	0.567	0.631
SVM^d^	0.814 (0.7734‐0.8539)		0.764	0.696	0.799	0.669
KNN^e^	0.781 (0.7383‐0.8230)		0.697	0.756	0.666	0.630
Random Forest	0.759 (0.7137‐0.8047)		0.725	0.655	0.762	0.620
Extremely Randomized Trees	0.735 (0.6894‐0.7813)		0.672	0.762	0.625	0.614
XGBoost	0.832 (0.7943‐0.8695)		0.752	0.708	0.821	0.690
LGBM^f^	0.831 (0.7928‐0.8693)		0.776	0.732	0.799	0.691
Gradient Boosting	0.838 (0.8012‐0.8750)		0.796	0.685	0.854	0.697
AdaBoost	0.815 (0 7761‐0.8536)		0.768	0.589	0.861	0.635
MLP^g^	0.830 (0.7921‐0.8671)		0.772	0.649	0.836	0.661

a AUC: Area Under the receiver operating characteristic curve.

b F1 Harmonic mean of precision and recall.

c LR: Logistic Regression.

d SVM: Support Vector Machine.

e KNN: K-Nearest Neighbors.

f LGBM: Light Gradient Boosting Machine.

g MLP: Multilayer Perceptron.

**Figure 5.**
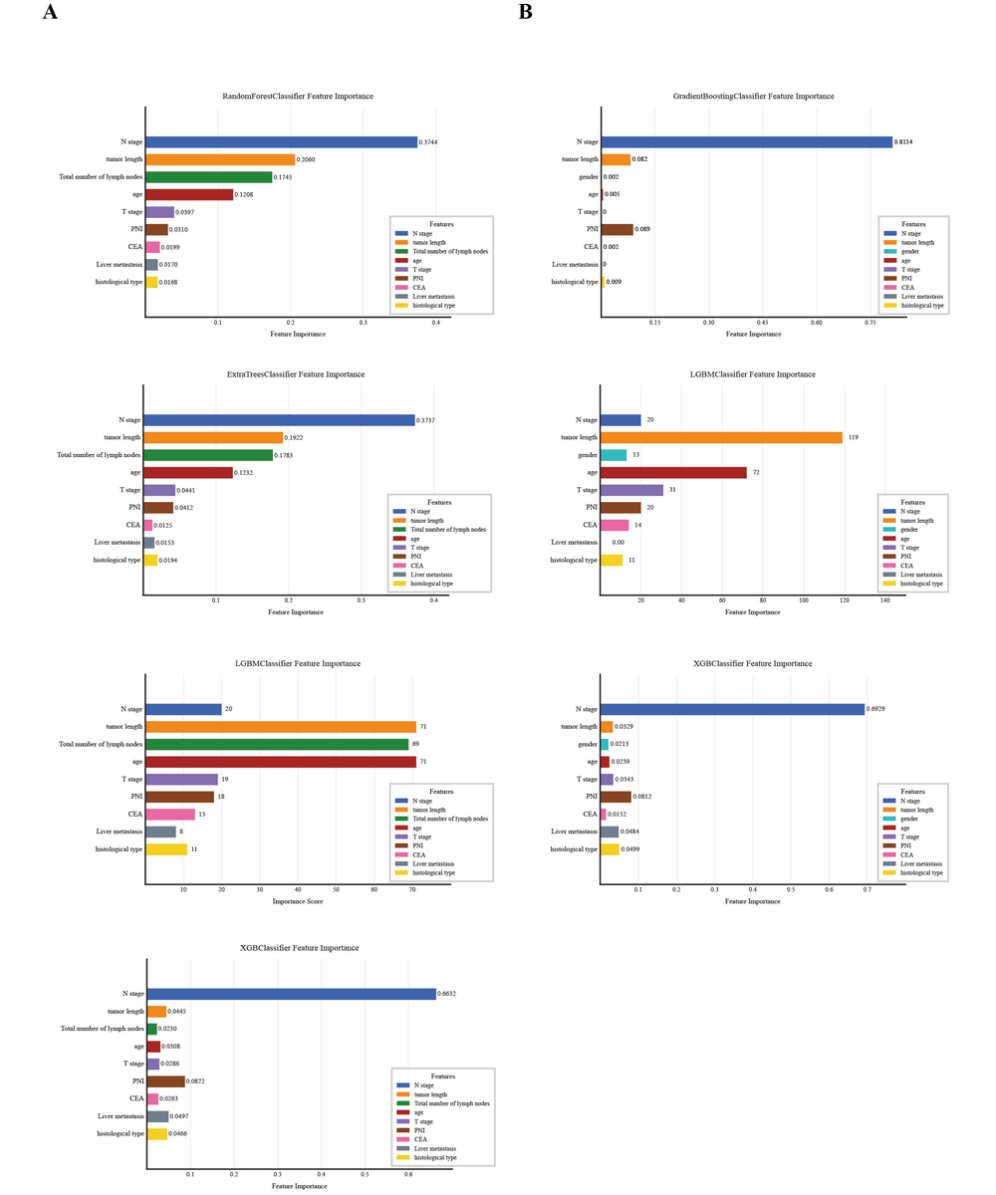
Rank the relative importance of each input variable. (A) Internal validation cohort and (B) external validation cohort. CEA: carcinoembryonic antigen; LGBM: Light Gradient Boosting Machine; PNI: perineural invasion; XGB: XGBoost**.**

**Figure 6.**
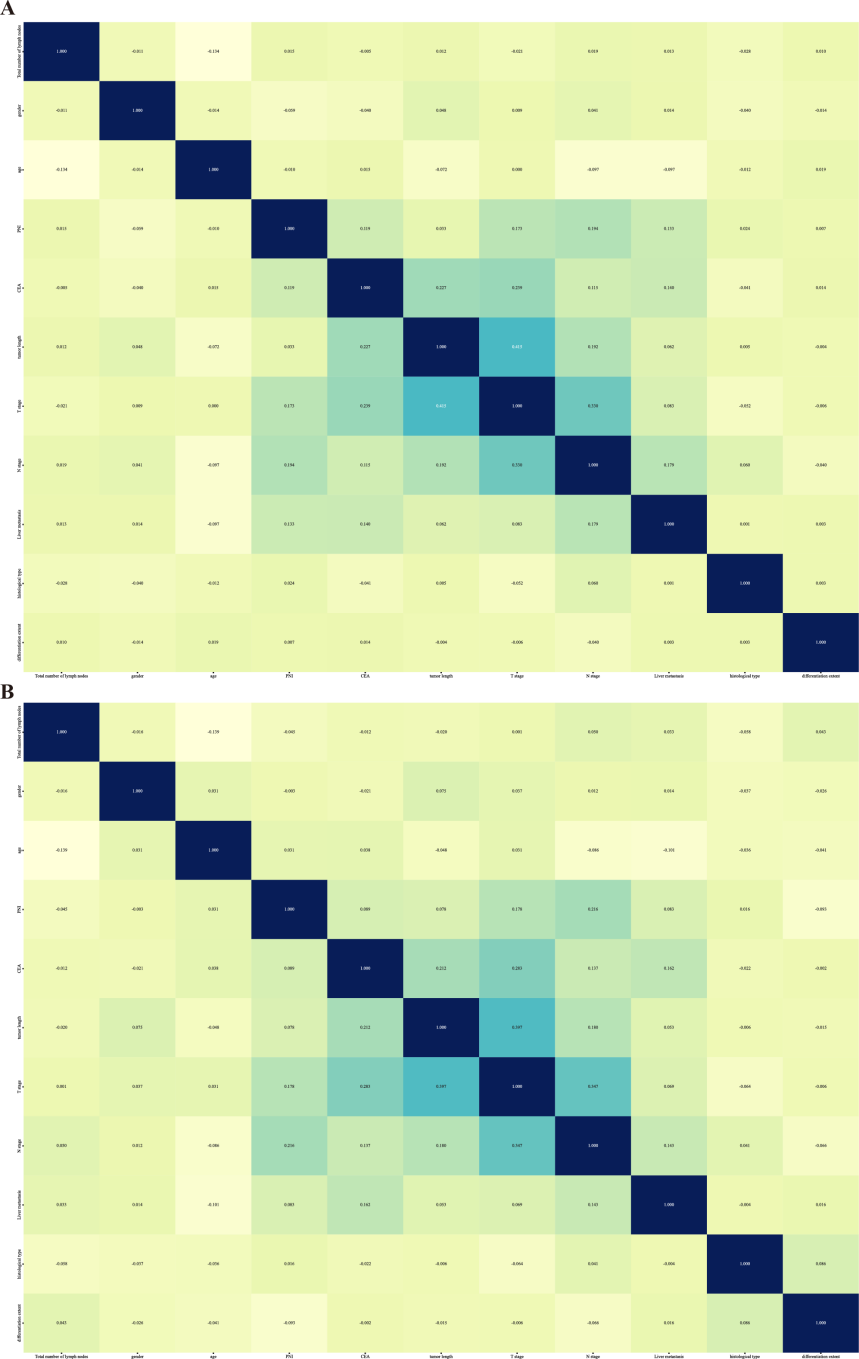
Correlation of clinical features. (A) Internal validation cohort and (B) external validation cohort.

The relative importance of model variables varies between the internal and external validation sets, depending on the characteristics. In the internal validation set, the clinical N-stage is the most important variable for the RF, ET, and XGB models. However, for the LGBM model, age and tumor length are the most important variables. In the external validation set, tumor length, clinical N-stage, and nerve invasion are among the top three important features for both the GB and LGBM models (Figure 5). We assessed feature correlations using a heatmap (Figure 6). In both validation sets, no significant correlations were observed, indicating the absence of collinearity and the independence of variables.

### Calibration Curve Analysis

**Figure 7.**
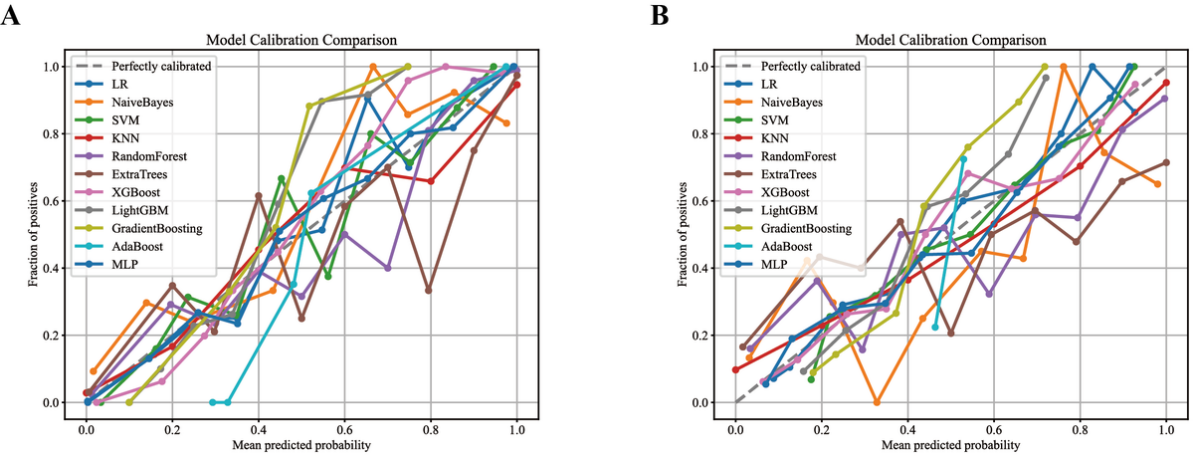
Calibration curve of lymph node metastasis prediction in the internal validation cohort (A) and external validation cohort (B) of patients. The dashed diagonal line represents perfect calibration where predicted probability equals actual probability. KNN: K-Nearest Neighbors; LR: Logistic Regression; MLP: Multilayer Perceptron; SVM: Support Vector Machine.

Calibration curves (Figure 7) demonstrated the agreement between predicted probabilities and observed event frequencies. In the internal cohort (Figure 7A), the XGB curve adhered closest to the ideal diagonal (45° reference line) throughout, with minimal deviation from actual frequencies in the 0.3‐0.7 probability range; ET exhibited consistent positioning above the diagonal at >0.8 probabilities (systematic overestimation), while RF deviated slightly below the diagonal between 0.6 and 0.8 (mild underestimation). In the external cohort (Figure 7B), XGB maintained the smallest overall deviation (closest to the diagonal); GB showed systematic distribution below the diagonal at 0.4‐0.6 probabilities (underestimation), and LGBM positioned above the diagonal at <0.3 thresholds (overestimation).

### Decision Curve Analysis

**Figure 8.**
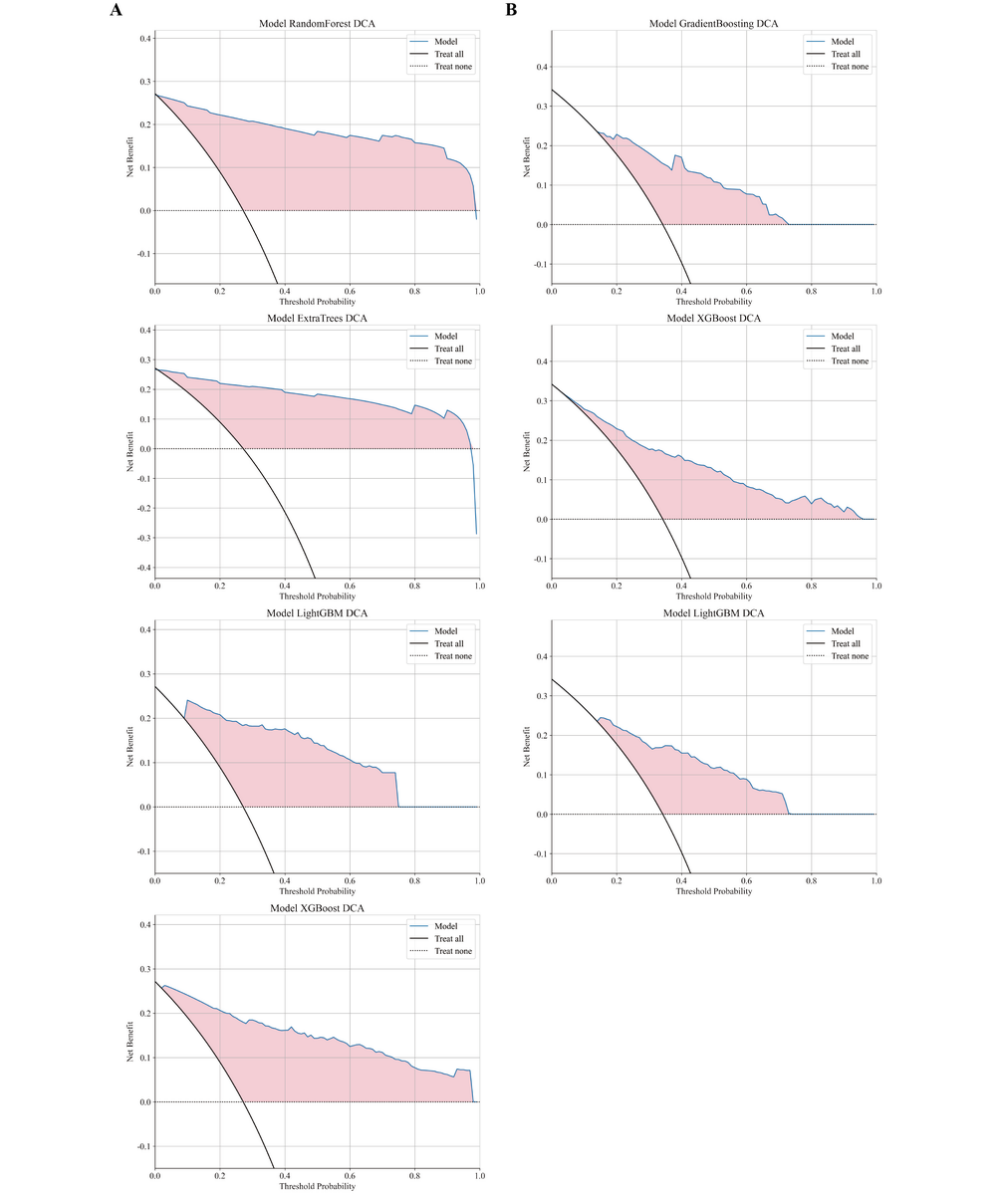
Decision curve analysis of lymph node metastasis prediction in the internal validation cohort (A) and external validation cohort (B) of patients with RC. DCA: decision curve analysis.

We evaluated the net benefits of the models in both validation sets using DCA (Figure 8). In the internal validation set, DCA indicates that the XGB model has a net benefit similar to that of the XGB and LGBM models at lower threshold probabilities (approximately 0‐0.3). Furthermore, the XGB model maintains net benefit across a broader range of threshold probabilities (approximately 0‐0.9). Between 0.7 and 0.9, where the net benefits of other models decrease, the net benefit of the XGB model still sustains. The RF model shows net benefit within the range of 0‐0.8. The ET model has a net benefit from 0.1 to 0.75. The LGBM model demonstrates net benefit in the range of 0.1‐0.7. In the external validation set, DCA reveals that the XGB model’s net benefit is higher than that of the GB and LGBM models when the threshold probability is between 0.3 and 0.4. Furthermore, the XGB model maintains net benefit across a broader range of threshold probabilities (approximately 0‐0.4). The GB model shows net benefit within the range of 0.1‐0.3, while the LGBM model has net benefit from 0.1 to 0.35.

#### Final Model Selection

XGB was selected as the definitive predictive model through comprehensive evaluation of cross-cohort discriminative stability, calibration fidelity, and clinical utility. The model achieved AUC values of 0.942 (95% CI 0.925‐0.959) in the internal cohort and 0.832 (95% CI 0.794‐0.869) in the external cohort, with a reduction in AUC of -0.110 between cohorts. This reduction was smaller than those observed for RF (−0.205), ET (−0.229), and GB (−0.071). Calibration curves demonstrated closest alignment to the ideal diagonal in the internal cohort (minimal deviation at 0.3‐0.7 probabilities), whereas ET systematically overestimated risk at probabilities >0.8 and RF underestimated at 0.6‐0.8. In the external cohort, XGB maintained minimal deviation, GB underestimated at 0.4‐0.6 probabilities, and LGBM overestimated at thresholds <0.3. DCA revealed that XGB sustained the broadest net benefit range (0‐0.9) in the internal validation set, outperforming comparison models at 0.7‐0.9 thresholds. In the external set, it maintained net benefit across 0‐0.4 thresholds and yielded higher net benefit than GB and LGBM at 0.3‐0.4 probability thresholds.

#### Confusion Matrix Analysis

Multimedia Appendix 4 displays the confusion matrices of the XGB model in the internal (A) and external (B) validation cohorts. In the internal cohort (n=500), the model correctly identified 333 LNM-negative cases (true negative) and 107 metastasis-positive cases (true positive), with 32 false positive and 28 false negative (FN) predictions. In the external cohort (n=500), it detected 160 true negative and 216 true positive cases, while generating 35 false positive and 89 FN classifications. The positive predictive value was 77% (107/139) in the internal cohort and 86.1% (216/251) in the external cohort; the negative predictive values were 92.2% (333/361) and 64.3% (160/249), respectively.

## Discussion

### Principal Findings

The main findings demonstrate that the XGB model achieved optimal performance in predicting RC LNM, with tumor differentiation grade, clinical T stage, tumor length, neural invasion, N stage, and total lymph node count identified as independent predictors. RC LNM significantly impacts patient prognosis, necessitating accurate prediction of LNM for the development of effective treatment plans. While traditional diagnostic methods offer some insights, their accuracy is constrained. This study employed ML algorithms to develop 11 prediction models for RC LNM. We extracted clinical data from the SEER database for the training and internal validation sets. Additionally, we acquired relevant clinical data from CT scans at the author’s hospital, utilizing precise pixel-level annotation and measurement for the external validation set. We filtered clinical data using the LASSO, univariate, and multivariate logistic regression analyses to develop and validate a predictive model for LNM. The selection of the optimal model was determined through comparative analysis of internal and external validation sets, leveraging the AUC, calibration curves, and DCA for precise assessment.

In this study, we successfully developed and validated 11 ML models for predicting LNM in RC. This study revealed three key findings. First, the six independent predictors of LNM include tumor differentiation, clinical T stage, N stage, tumor length, total number of lymph nodes, and neural invasion. Second, among the 11 constructed ML models for predicting LNM, 10 models had an AUC greater than 0.9 in the internal validation set and 7 models had an AUC greater than 0.80 in the external validation set. Lastly, following a comparison of the models’ performance and their validation set discrepancies, the XGB model emerged as the most suitable among the 11 models.

### Comparison With Prior Studies

Utilizing LASSO logistic regression and multivariate analysis, we identified tumor differentiation, clinical T stage, N stage, tumor length, neural invasion, and total number of lymph nodes as the key clinical predictive factors. Low tumor differentiation, advanced clinical T stage, advanced N stage, increased tumor length, and neural invasion are significantly associated with LNM. Identifying these factors is essential to comprehend the tumor’s biological behavior and invasive potential. Variations in feature importance across models (Figure 5) arise from the following factors: First, clinical data encompass both continuous (eg, tumor length) and categorical variables (eg, pathological grade), where XGB’s exact splitting favors continuous features while LGBM’s histogram-based algorithm optimizes high-dimensional sparse feature processing. Second, regularization differences lead to distinct weight allocation—XGB’s combined L1/L2 regularization strictly controls overfitting, whereas LGBM’s leaf-wise growth prioritizes locally significant features. Third, although LASSO-based feature selection reduced dimensionality, residual feature correlations are differentially processed. These inherent algorithmic variations confirm that no single model can fully capture feature relationships, necessitating multimodel comparison for complementary insights. Poorly differentiated tumor cells show greater invasiveness and a higher likelihood of LNM. A study by Saraste et al [24] involving 1664 patients with RC from the Swedish Rectal Cancer Registry between 2007 and 2010 concluded through multivariate analysis that poorly differentiated tumors are a significant risk factor for LNM, aligning with the findings of this study. Advanced clinical T stage is a direct indicator of deeper tumor invasion. A study by von den Grün et al [25], which involved a binary logistic regression multivariate analysis of 776 patients with RC, revealed that advanced T stage is a significant prognostic factor for LNM in RC. Previous studies [25-27] have established that clinical T stage and N stage significantly influence the prognosis and treatment of RC, aligning with the results of the univariate and multivariate analyses presented in Table 2. Increased tumor length may afford tumor cells increased opportunities to interact with adjacent lymph nodes. In a study [28] that employed 7 clinical parameters as independent prognostic factors to develop a nomogram prediction model, 6484 patients with RC from the SEER database were analyzed using Cox proportional hazards regression. This analysis identified independent prognostic factors such as T stage and tumor length. However, unlike this study, the aforementioned research did not include an external validation cohort. The presence of neural invasion indicates a high level of tumor invasiveness and the potential for dissemination along neural pathways. Neural invasion is a significant factor in both univariate and multivariate analyses, as well as in evaluating the importance of model features. Studies by Ueno et al [29] and Song et al [30] have shown that neural invasion is a significant factor in LNM in RC. The total number of perirectal examined lymph nodes, as a key factor, plays a crucial role in accurate staging and prognostic assessment of RC. Guan et al [31] found that a higher number of perirectal examined lymph nodes in RC is linked to more accurate lymph node staging and improved survival. Analyzing data from stage I to III resected RC in multi-institutional Chinese and US SEER databases revealed that an increasing number of perirectal examined lymph nodes significantly raises the proportion of cases shifting from lymph node-negative to lymph node-positive. Furthermore, after adjusting for confounding factors, overall survival consistently improves.

Utilizing the selected clinical characteristics, we developed a predictive model for LNM in RC. We extracted a cohort of 2454 patients with RC from the SEER database and randomly selected 500 cases for the internal validation cohort. The remaining 1954 cases served as the training cohort, while an additional 500 cases from the author’s hospital constituted the external validation cohort. In the internal validation set, the RF and ET models achieved an AUC of 0.964. The LGBM and XGB models also performed remarkably well, with AUC values of 0.943 and 0.942, respectively. Among all models, the NB model had the lowest AUC of 0.859, while the remaining 10 models all exceeded 0.9. In the external validation set, the GB model demonstrated the best performance with an AUC of 0.838, followed by the XGB and LGBM models, which reached AUC values of 0.832 and 0.831, respectively. In the study by Guan et al [18], 6578 patients with RC were enrolled across several institutions, including the Cancer Hospital of the Chinese Academy of Medical Sciences, Peking Union Medical College, Changhai Hospital of Naval Medical University, and the Second Affiliated Hospital of Harbin Medical University. The XGB model was identified as the optimal in their study, achieving AUCs of 0.78 and 0.71 across two validation cohorts. In contrast, our study’s XGB model showed superior performance, achieving an AUC of 0.942 in the internal validation cohort and 0.832 in the external validation cohort. In the external validation cohort, LNM status, clinical T and N stages, and tumor length were ascertained through the annotation of CT imaging combined with clinical data. However, across both internal and external validation cohorts, most models in this study demonstrated relatively good performance across various validation sets, with the AUCs of the optimal models all exceeding 0.80, indicating a certain level of accuracy and reliability. These results demonstrate that our models exhibit high stability and generalizability across diverse datasets. Regardless of whether the patients with RC are in the United States or China, our models show accurate predictive ability for forecasting LNM in RC. Second, the LNM status and associated details in the 500 case records from the hospital were meticulously annotated by 50 physicians. These annotations were made by integrating clinical and pathological data using imaging software on enhanced CT scans. Following the initial annotation, two radiologists, possessing 8 and 20 years of experience, respectively, conducted a review and confirmation of the annotations. The multi-physician annotation strategy, which incorporates clinical pathology data and imaging software, boosts the reliability of the data and, in turn, elevates the predictive accuracy of the model. A similar approach was taken in Liu et al’s study [32], wherein integrating clinical data with ML for magnetic resonance image analysis elevated the AUC value of single-region radiomics from 0.702 to 0.827. In a study by Wan et al [33], an automated segmentation method using deep learning demonstrated potential for predicting LNM in RC. However, our manual annotation method ensures precise localization of small lymph nodes (diameter < 5 mm) and ambiguous lesions, which are crucial for RC staging but challenging for automated tools. This strategy is particularly valuable in resource-limited environments lacking artificial intelligence infrastructure. Although magnetic resonance imaging is widely regarded as the gold standard for local T-staging and lymph node status assessment in RC, in this study, we chose to analyze CT images due to the diversity of data sources and the prevalence of CT images. CT scanning is less costly and quicker. Particularly, in resource-limited medical settings, CT remains a routine examination tool that can effectively provide important information on lymph node status and tumor staging. This highlights the benefits of integrating both clinical data and imaging information into ML models. While the annotation of CT images and their incorporation are standard practices in traditional radiomics and the development of deep learning models, they are less common in models derived solely from clinical data. This study shows that employing imaging software to annotate CT scans and extract data for clinical predictive models not only yields high-quality data for model development but also presents an innovative approach to data acquisition and processing for future studies.

### Strengths and Limitations

A key innovation of this study is the comprehensive utilization of diverse advanced ML algorithms, coupled with the validation of the model’s performance across data from various sources. Our models have shown strong generalization capabilities, as evidenced by their performance in both internal and external validation cohorts. Additionally, using LASSO logistic regression for feature selection enabled us to pinpoint key clinical predictive factors. This approach not only bolstered the model’s predictive accuracy but also improved its interpretability. We presented the importance of features in a ranked order and investigated the correlations among variables with a correlation heatmap. Finally, the optimal model was ascertained through a comprehensive, evidence-based methodology integrating multimetric performance evaluation (AUC, 95% CI, accuracy, sensitivity, specificity, and *F*_1_-score), augmented by high-resolution assessment of calibration curves and DCA.

Despite strong performance in both the internal and external validation sets, our model in this study has some limitations. First, the dataset is constrained by its population representativeness. The SEER database comprises exclusively American cases, and the dataset from the author’s hospital consists solely of Chinese cases. This could impact the model’s ability to generalize across diverse populations. In Shulman et al.’s study [34], which analyzed 34,500 patients with RC, it was found that patients of different races exhibited varying lymph node statuses. Second, the model did not include variables like BMI, alpha-fetoprotein, cancer antigen 125, carbohydrate antigen 19-9 [18], which were previously explored in clinical information. This omission could impact the model’s predictive accuracy and comprehensiveness. Future studies could further refine predictive accuracy by incorporating serum biomarkers (eg, BMI, carbohydrate antigen 19-9) and emerging indicators such as circulating tumor DNA, combined with radiomic features. Third, FN classifications warrant clinical attention. The FN rate was 20.7% (28/135) in the internal cohort and 29.2% (89/305) in the external cohort. Such misclassifications may lead to undertreatment (eg, omission of adjuvant chemotherapy or lymph node dissection), increasing risks of recurrence and metastasis. Crucially, while our model was developed to surpass conventional CT assessments, reducing FNs remains a priority for future optimization through biomarker integration or advanced imaging techniques. The study by Yu et al [35] demonstrated that incorporating multicenter data and multimodal features significantly reduced FN rates, providing a feasible direction for refining our model. In this study, we utilized 11 ML methods. Future research could further enhance the model’s predictive performance by integrating diverse ML algorithms and leveraging their unique strengths.

### Conclusions

In conclusion, this study successfully developed a ML-based risk prediction model for LNM in RC, validating its performance using both an internal and an external validation set. Through the analysis of extensive clinicopathological data, we identified tumor differentiation, clinical T stage, N stage, tumor length, neural invasion, and total number of lymph nodes as independent predictive factors. Among the 11 models evaluated, the XGB model demonstrated the best predictive performance. These models are anticipated to aid in clinical decision-making, offering vital insights for treatment selection and prognostic assessment of patients with RC. They hold significant clinical utility and scientific importance. Concurrently, the external validation set demonstrates that clinical data derived from CT imaging annotation and measurement are comparable to traditionally obtained clinical data and can effectively serve as a source of clinical data for ML applications.

## Supplementary material

10.2196/73765 Multimedia Appendix 1 Normalization standards of clinical data.

10.2196/73765 Multimedia Appendix 2 The hyperparameter settings and tuning strategies of the 11 models.

10.2196/73765 Multimedia Appendix 3 Range standard for property values of clinical features in models.

10.2196/73765 Multimedia Appendix 4 Confusion matrices for lymph node metastasis (LNM) prediction in rectal cancer (RC) patients.

## Abbreviations

AB: Ada-Boost
AJCC: American Joint Committee on Cancer
AUC: area under the receiver operating characteristic curve
CEA: carcinoembryonic antigen
CT: computed tomography
DCA: decision curve analysis
ET: Extremely Randomized Trees
GB: Gradient Boosting
KNN: K-Nearest Neighbors
LASSO: Least Absolute Shrinkage and Selection Operator
LGBM: Light Gradient Boosting Machine
LNM: lymph node metastasis
LR: Logistic Regression
ML: machine learning
MLP: Multilayer Perceptron
NB: Naive Bayes
PNI: Perineural Invasion
RC: rectal cancer
RF: Random Forest
SEER: Surveillance, Epidemiology, and End Results
SVM: Support Vector Machine
XGBoost: Extreme Gradient Boosting
