# Supplementary material for: Predicting Lymph Node Metastasis in Rectal Cancer: Development and Validation of a Machine Learning Model Using Clinical Data
Source: JMIR Med Inform. 2025 Sep 23;13:e73765. doi: 10.2196/73765 (PMC12456929; doi:10.2196/73765)
Supplement: Multimedia Appendix 1 [file medinform-v13-e73765-s001.docx]

**Supplement Table S1. Normalization standards of clinical data.**

| **Clinical data** | **Normalization Standard** |
| --- | --- |
| **Basic demographic data** |  |
| Conflict of interest | Conflict of interest |
| Conflict of interest | Conflict of interest |
| Conflict of interest | Conflict of interest |
| Conflict of interest | Conflict of interest |
| Conflict of interest | Conflict of interest |
| Conflict of interest | Conflict of interest |
| Conflict of interest | Conflict of interest |
| External validation | Imaging Comparison |
| **PNI** |  |
| Training cohort and Internal validation | Pathological examination |
| External validation | Imaging studies |
| **Laboratory index** |  |
| CEA | Positive (>5ng/ml) |
|  | Negative (<5ng/ml) |
| **Clinical T stage** |  |
| Training cohort and Internal validation | Pathological examination amd Imaging studies |
| External validation | Pixelated annotation of imaging. |
| **Clinical N stage** |  |
| Training cohort and Internal validation | Pathological examination amd Imaging studies |
| External validation | Pixelated annotation of imaging. |

**Abbreviation:** PNI,perineural invasion; CEA, carcinoembryonic antigen.
