# Supplementary material for: Predicting Lymph Node Metastasis in Rectal Cancer: Development and Validation of a Machine Learning Model Using Clinical Data
Source: JMIR Med Inform. 2025 Sep 23;13:e73765. doi: 10.2196/73765 (PMC12456929; doi:10.2196/73765)
Supplement: Multimedia Appendix 2 [file medinform-v13-e73765-s002.docx]

The hyperparameter Settings and tuning strategies of the 11 models.

Logistic Regression (LR) employed no regularization penalty (penalty='none') with maximum iterations capped at 100; Support Vector Machine (SVM) enabled probability output using a linear kernel (kernel='linear') and maximum iterations of 100; Random Forest utilized 5 estimators, maximum depth of 3, and minimum samples split of 4; XGBoost configured 6 estimators with binary logistic objective function (objective='binary:logistic'), maximum depth 3, minimum child weight 0.2, while disabling label encoder and using error rate as the evaluation metric; LightGBM implemented 2 estimators, maximum depth 1, and minimum child weight 0.5; Extra Trees employed 60 estimators, maximum depth 5, and minimum samples split 2; both Gradient Boosting and AdaBoost used 10 estimators; Multilayer Perceptron (MLP) adopted a specific hidden layer architecture (61-128-64-32) with 300 maximum iterations and stochastic gradient descent solver. For Naive Bayes and K-Nearest Neighbors (KNN), the former maintained default Gaussian parameters while the latter optimized neighbors to 5 (n_neighbors=5) through preliminary experiments.

A three-phase tuning framework was implemented: First, Bayesian optimization with 50 iterations explored core parameter spaces (e.g., depth ranges for tree-based models), establishing initial search boundaries. Second, grid search refined performance-sensitive models (XGBoost, LightGBM, MLP), focusing on critical parameter interactions (e.g., learning_rate × subsample for XGBoost). Finally, linear models (LR, SVM) underwent 10-fold cross-validation for precision regularization tuning of parameter C. Notably, tree-based models (Random Forest, Extra Trees, Gradient Boosting) restricted estimator counts to 5-60 - a design preventing overfitting in the low-dimensional feature space (final dimensionality=13), with validation showing 0.3% AUC drop when Random Forest estimators exceeded 5. Dynamic estimator adjustment via early stopping (patience=50 rounds) optimized tree counts for XGBoost and LightGBM, while Naive Bayes and KNN maintained default configurations due to absence of critical tunable parameters. Complete search spaces and selection criteria are documented in Supplementary Table S2, including grid search details for key parameters such as XGBoost's learning rate (0.01-0.1) and subsample ratio (0.6-1.0).
