# Supplementary material for: Predicting Lymph Node Metastasis in Rectal Cancer: Development and Validation of a Machine Learning Model Using Clinical Data
Source: JMIR Med Inform. 2025 Sep 23;13:e73765. doi: 10.2196/73765 (PMC12456929; doi:10.2196/73765)
Supplement: Multimedia Appendix 3 [file medinform-v13-e73765-s003.docx]

**Supplement Table S2. Range standard for property values of clinical features in models.**

| **Variables** | **Property Values** |
| --- | --- |
| **Sex** |  |
| Male | **0** |
| Female | **1** |
| **Age** |  |
| 20≤X≤24 | **0** |
| 25≤X≤29 | **1** |
| 30≤X≤34 | **2** |
| 35≤X≤39 | **3** |
| 40≤X≤44 | **4** |
| 45≤X≤49 | **5** |
| 50≤X≤54 | **6** |
| 55≤X≤59 | **7** |
| 60≤X≤64 | **8** |
| 65≤X≤69 | **9** |
| 70≤X≤74 | **10** |
| 75≤X≤79 | **11** |
| 80≤X≤84 | **12** |
| 85≤X | **13** |
| **Total number of lymph nodes** |  |
| 0<X≤5 | **0** |
| 5<X≤10 | **1** |
| 10<X≤15 | **2** |
| 15<X≤20 | **3** |
| 20<X≤25 | **4** |
| 25<X≤30 | **5** |
| 30<X | **6** |
| **Tumor length** |  |
| 0<X≤5 | **0** |
| 5<X≤10 | **1** |
| 10<X | **2** |
| **PNI** |  |
| Negative | **0** |
| Positive | **1** |
| **CEA** |  |
| Negative | **0** |
| Positive | **1** |
|  |  |
| **Clinical T stage** |  |
| T1 | **0** |
| T2 | **1** |
| T3 | **2** |
| T4 | **3** |
| **Clinical N stage** |  |
| N0 | **0** |
| N1 | **1** |
| N2 | **2** |
| **Liver metastasis** |  |
| Negative | **0** |
| Positive | **1** |
| **Histological type** |  |
| Adenocarcinoma | **0** |
| Mucinous/Signet-ring cell | **1** |
| others | **2** |
| **Differentiation extent** |  |
| Well differentiated; Grade I | **0** |
| Moderately differentiated; Grade II | **1** |
| Poorly/Undifferentiated differentiated; Grade III | **2** |

**Abbreviation:** PNI,perineural invasion; CEA, carcinoembryonic antigen.
