# Supplementary figures and images for: Predicting Lymph Node Metastasis in Rectal Cancer: Development and Validation of a Machine Learning Model Using Clinical Data
Source: JMIR Med Inform. 2025 Sep 23;13:e73765. doi: 10.2196/73765 (PMC12456929; doi:10.2196/73765)

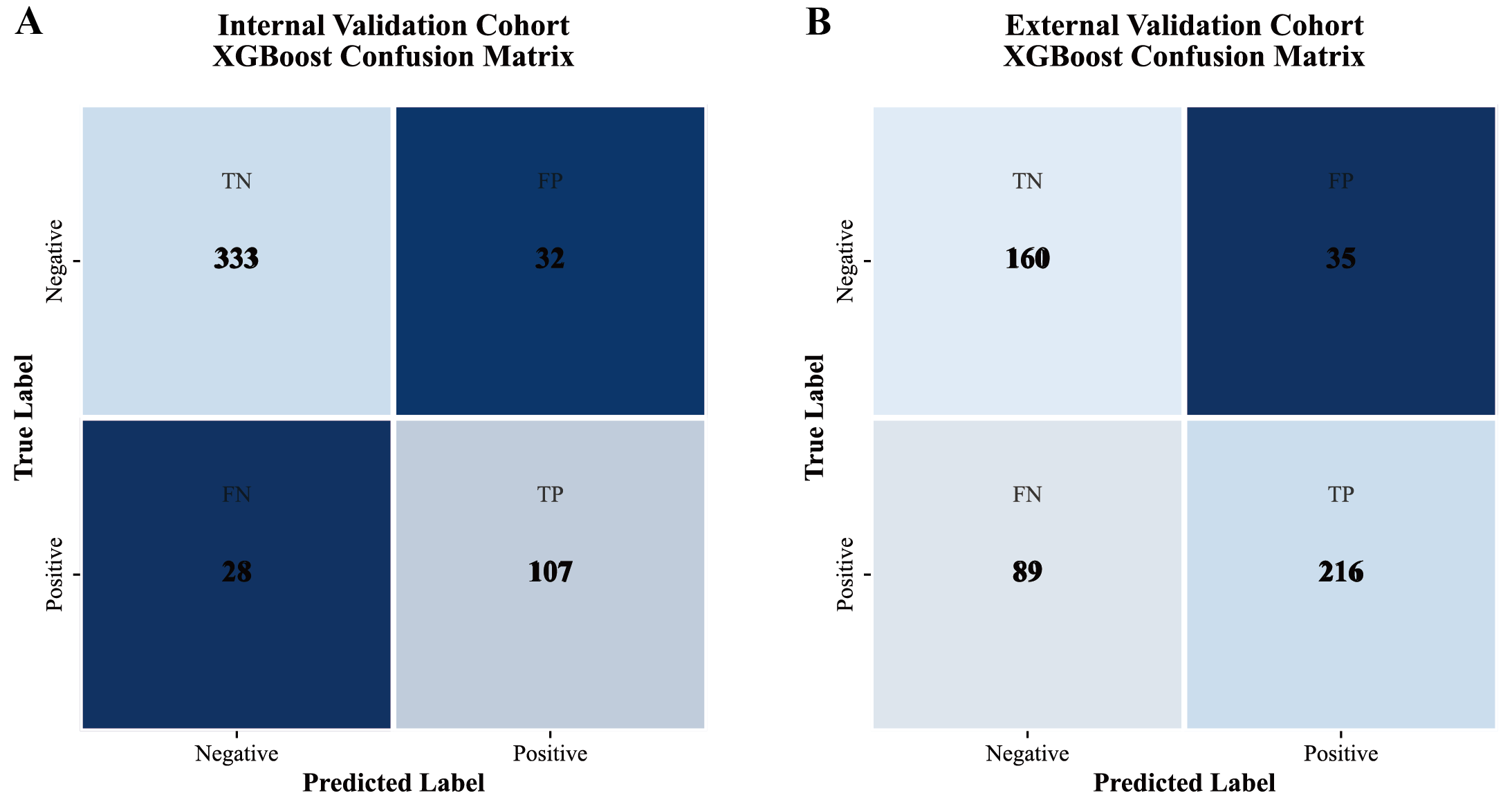

Supplement: Multimedia Appendix 4 [file medinform-v13-e73765-s004.png]
